# Memory discrimination is promoted by the expression of the transcription repressor WT1 in the dentate gyrus

**DOI:** 10.3389/fnbeh.2023.1130840

**Published:** 2023-09-27

**Authors:** Leonardo Munari, Vishwendra Patel, Nicholas Johnson, Chiara Mariottini, Som Prabha, Robert D. Blitzer, Ravi Iyengar

**Affiliations:** Department of Pharmacological Sciences and Institute for Systems Biomedicine, Icahn School of Medicine at Mount Sinai, New York, NY, United States

**Keywords:** dentate gyrus, memory discrimination, WT1 = wilm’s tumor 1, hippocampus, cell excitability, DREADD, CA3, memory

## Abstract

The hippocampus is critical for the precise formation of contextual memories. Overlapping inputs coming from the entorhinal cortex are processed by the trisynaptic pathway to form distinct memories. Disruption in any step of the circuit flow can lead to a lack of memory precision, and to memory interference. We have identified the transcriptional repressor Wilm’s Tumor 1 (WT1) as an important regulator of synaptic plasticity involved in memory discrimination in the hippocampus. In male mice, using viral and transgenic approaches, we showed that WT1 deletion in granule cells of the dentate gyrus (DG) disrupts memory discrimination. With electrophysiological methods, we then identified changes in granule cells’ excitability and DG synaptic transmission indicating that WT1 knockdown in DG granule cells disrupts the inhibitory feedforward input from mossy fibers to CA3 by decreasing mIPSCs and shifting the normal excitatory/inhibitory (E/I) balance in the DG → CA3 circuit in favor of excitation. Finally, using a chemogenetic approach, we established a causal link between granule cell hyperexcitability and memory discrimination impairments. Our results suggest that WT1 enables a circuit-level computation that drives pattern discrimination behavior.

## Introduction

Episodic memories (related to “what, when, where”) are essential for survival as they allow individuals to predict future events. From an evolutionary standpoint, the memory system is efficiently optimized to anticipate the location of resources in the environment, avoid danger, and guarantee the reproduction and survival of the species (Allen and Fortin, 2013). Despite a high degree of similarity between episodic memories, individuals can remember the distinct characteristics of different yet related memories–for instance, discrete events in familiar places or similar routines. However, in some cases experiences involving similar components overlap, causing memory interference and resulting in a spurious association between discrete events, so-called “false memories” (Ramirez et al., 2013). Memory interference is a major limitation in memory capacity, and it is thought to be a key cognitive symptom in several neuropsychiatric conditions, such as schizophrenia, autism, and mood disorders (Shelton and Kirwan, 2013; Das et al., 2014; Engin et al., 2015).

The hippocampus plays a critical role in consolidation and recalling of episodic memories (Bird and Burgess, 2008). The dentate gyrus (DG) is the first stage of the hippocampal trisynaptic circuit that receives input from the entorhinal cortex and performs pattern separation, the process by which similar inputs from the entorhinal cortex are transformed into more differentiated DG outputs (Gluck et al., 2003; Leutgeb et al., 2007; Madroñal et al., 2016). Then, DG granule cells (DGCs) relay information to CA3 pyramidal neurons, directly through excitatory synapses and indirectly through inhibitory interneurons. The recruited ensembles of pyramidal neurons and interneurons are thought to encode and retrieve selective episodic memories (Tonegawa et al., 2015). Recent data suggest that the DG → CA3 recruitment of inhibition shapes the overall mossy fiber input to CA3 and enhances precision during memory recall (Guo et al., 2018). The mossy fiber synapses onto CA3 interneurons are rapidly strengthened by granule cell activity (Neubrandt et al., 2018), and this enhancement correlates with filopodial growth, improved contextual discrimination, and prolonged memory retention for spatial tasks (Mori et al., 2004; Ruediger et al., 2011; Park et al., 2016; Guo et al., 2018). Understanding how the neural circuits within the DG → CA3 network mediate memory precision has received much interest, yet the precise cellular and molecular mechanisms remain elusive.

The transcriptional repressor Wilm’s Tumor 1 (WT1) has been studied primarily in the context of urogenital development (Rauscher, 1993; Fischbach et al., 2005; Klattig et al., 2007), while its function in normal brain physiology and memory has received less attention and is still poorly understood. A few studies reported a role for WT1 in neurodegeneration associated with Alzheimer’s disease (Lovell et al., 2003; Wang et al., 2020) and depressive-like behaviors (Ji et al., 2020). We have previously identified WT1 as a key molecular driver in memory formation during hippocampus-dependent learning tasks. In particular, forebrain WT1-deficient mice show reduced performances in fear extinction, reversal, and sequential learning tasks, revealing impaired memory flexibility. Further, an acute increase of WT1 levels in the CA1 region, the main hippocampal output, results in impaired memory consolidation (Mariottini et al., 2019). At the cellular level, acute ablation of WT1 in CA1 increases pyramidal neurons synaptic efficiency and excitability and disrupts the capability of these cells to integrate signals originating from the trisynaptic pathway with direct input from the entorhinal cortex (Mariottini et al., 2019).

Here, we have studied the role of WT1 in the dentate gyrus, and its contribution to memory discrimination for two similar contexts. Our findings demonstrate that ablation of WT1 in the DGCs impairs pattern separation, resulting in memory interference in a context discrimination test. Using chemogenetic approaches we causally established that pattern discrimination was rescued by selective silencing of DGCs. Together, these findings indicate that WT1 in DGCs controls feedforward inhibition to enable pattern separation.

## Materials and methods

### Animals

All animal experiments were performed according to ethical regulations and protocols approved by the Institutional Animal Care and Use Committee (IACUC) at Icahn School of Medicine at Mount Sinai.

#### Generation of the WT1 dentate gyrus granule cell knock-out mice (Wt1Δ*^DGC^*)

We have maintained an in-house colony where Wt1*^fl/fl^* mice which were originally obtained from Dr. Vicki Huff (Gao et al., 2006) and were crossed with CamKII-Cre mice [B6.Cg-Tg(Camk2a-cre)T29-1Stl/J; Jax Lab Stock# 005359] to generate Wt1Δ mice (Mariottini et al., 2019). Briefly, Wt1Δ mice express in the forebrain the Cre recombinase which results in the in-frame deletion of exons 8 and 9 and generates a truncated allele (Wt1Δ) encoding a shortened WT1 protein lacking zinc finger domains 2 and 3 (Gao et al., 2006). To knock down Wt1 specifically in the granule cells of the dentate gyrus (Wt1Δ*^DGC^*), we inject AVV8-CamKII-Cre-GFP or AVV8-CamKII-Cre-mCherry (Addgene, USA) virus into DG of Wt1*^fl/fl^* mice which were Cre recombinase negative (Supplementary Figure 3A). For DREADD experiments mice were injected with a mix (1:1) of AAV8-hSyn-DIO-hM4D(Gi)-mCherry (Addgene) + AAV8-CamKII(0.4)-iCRE-WPRE (Vector Lab, USA).

Throughout the study, control wild-type littermates were indicated as *Control* and they comprise the following subgroups: Wt1^+/+^, Camk2a-Cre positive; Wt1^+/+^, Camk2a-Cre negative and Wt1*^fl/fl^*, Camk2a-Cre negative. These were grouped for both electrophysiology and behavior experiments since there were no statistically different results among the genotypes.

To genotype Controls and Wt1Δ animals we used the following primers (Mariottini et al., 2019):

LoxP allele: Primer LoxP Forward 5′ CCT TTT ACT TGG ACC GTT TG 3′ and Primer LoxP Reverse 5′ GGG GAG CCT GTT AGG GTA 3′.

Cre allele: Primer Cre Forward 5′ GCG GTC TGG CAG TAA AAA CTA TC 3′ and Primer Cre Reverse 5′ GTG AAA CAG CAT TGC TGT CAC TT 3′.

#### Stereotaxic surgery and viral injection

Mice were deeply anesthetized with a mixture of ketamine (10 mg/mL) and xylazine (1 mg/mL) i.p., and placed in a stereotaxic apparatus (Kopf, USA). DG coordinates were the following relative to Bregma: AP: −2.2; ML: ± 1.3; DV: −2.0. CA1 coordinates were the following: AP: −2.0; ML: ± 1.5; DV: −1.2. All viruses were bilaterally injected at a rate of 0.1 μL/min for a total volume of 0.5 μL per side using a microsyringe (Hamilton, USA). The needle remained at the target site for additional 7–10 min after the injection. Behavioral experiments were performed 4 weeks after the surgery.

### Behavior experiments

All behavior tests were performed during the light cycle (6 a.m.–6 p.m.) and mice were handled (1–2 min) for 3 days before experiments. Animals were allowed to habituate to the behavior room for at least 1 h before the test. To test our hypothesis that Wt1Δ*^DGC^* mice would show a deficit in discriminate highly similar contexts, we conducted a contextual discrimination test that involved encoding and retrieval of contradictory and overlapping memories.

#### Contextual discrimination test (short protocol)

The test was conducted using three distinct contexts (A, B, C) which were modifications of Med-Associates fear conditioning chambers (29.5 × 23.5 × 21 cm). Context A had a glossy white plastic floor insert and was scented with 1% acetic acid from a tray underneath as an olfactory cue, the back wall of the chamber was covered with red paper as a visual cue. Context B was similar in shape to Context A (high interference context) with a metal gridded floor and was scented with citric lemon essence as an olfactory cue from a tray underneath. No visual cue was attached to the walls. Context C was very different from contexts A and B (low interference context). Chamber had a white plastic floor covered with bedding, curved walls with visual cues, and scented with grapefruit essence in the tray underneath the floor as an olfactory cue. Mice were first placed into Context A (pre-shock neutral context) and were allowed to explore it for 10 min. Six or twenty-four hours later, animals were fear-conditioned in Context B (training shock context). They were first allowed to explore Context B for 2 min, after that animal received two foot-shocks 1 min apart (0.65 mA, 2 s), followed by 1 min of free exploration. Animals were then removed from the chamber and placed back into their home cages. On the next day, animals were placed back into Context A (post-shock) and Context C (new context), 2–3 h apart. Mice were counterbalanced between contexts A and C. Twenty-four hours later mice were placed again in Context B (no shocks). Freezing time to each context was calculated using Ethovision (Noldus) for a total of 5 min. We calculated the discrimination ratio: (freezing in Context A)/(freezing in Context A + freezing in Context B) to detect bi-directional shifts in the discrimination-generalization balance.

#### Contextual discrimination test (extended protocol)

The same contexts were used for the extended protocol. Animals explored contexts A and B exactly as in the short protocol. Two days after being fear conditioned in shock Context B, mice were placed for 6 consecutive days in contexts A and B, 2–3 h apart. Animals were counterbalanced. On day 7, mice were tested in the low interference Context C to investigate their ability to discriminate. They were again tested in contexts A and B on days 14 and 21 after being shocked in Context B. Freezing behavior to each context was calculated using Ethovision (Noldus) for a total of 5 min.

#### DREADD experiment

Animals underwent the context discrimination test (short protocol) Clozapine N-oxide (CNO, 1 mg/kg, i.p.) or saline were injected 45 min before mice were fear conditioned in Context B. Injection of CNO activated the hM4D inhibitory DREADD receptor and silenced DGCs (Supplementary Figure 4).

### Immunohistochemistry

Mice were deeply anesthetized with an overdose of ketamine (100 mg/mL) and transcardially perfused with cold PBS and then 4% paraformaldehyde (PFA) in PBS, followed by 48 h post-fixation in the same solution. Brains were sectioned to 40 μm using a vibratome (Leica VT 1000S, Germany), slices were incubated in 0.5% TritonX-100 PBS for 20 min before being blocked in a solution containing 10% normal goat serum (Vector), 0.25% TritonX-100 in PBS for at least for 2 h at room temperature. After that, primary antibodies were added to a solution containing 3% goat serum, 0.1% TritonX-100 in PBS, and incubated overnight at 4°C. Primary antibodies: mouse anti-mCherry (cat#125096, ABCAM, UK), rabbit anti-GAD2, rabbit anti-Lucifer Yellow (cat# A-5750, Invitrogen, USA). On the next day, slices were washed with PBS (3 × 10 min) and incubated with secondary antibodies coupled to either Alexa Fluor 488, Alexa Fluor 568, or Alexa Fluor 647 dyes (Invitrogen) for 2 h. Sections were then washed again in PBS (3 × 10 min), and Hoechst 33342 (1:10,000 dilution, Invitrogen) was used to label nuclei (15–20 min). Sections were mounted using ProLongTM Gold (Invitrogen) on glass slides. All imaging was done using Zeiss LSM 880 confocal microscope and image on 20× or 40× lenses.

### C-Fos+ cells quantification

Because the transcription factor c-Fos serves as an indirect marker of neuronal activity, we quantified the number of c-Fos + cells in mice either treated with saline or CNO (1 mg/kg, ip). WT1*^fl/fl^*, Cre negative male mice were stereotaxically injected into the DG with a mix (1:1) of AAV8-hSyn-DIO-hM4D(Gi)-mCherry (Addgene) + AAV8-CamKII(0.4)-iCRE-WPRE (Vector Lab) and tested 4 weeks later. Mice were injected with either saline (*n* = 3) or CNO (*n* = 4) and 45 min later fear conditioned in Context B (2 foot-shocks, 1 min apart, 0.65 mA, 2 s) and sacrificed 90 min later. Brains were removed, fixed and stained for c-Fos (antibody: Cell Signaling #31254) following our immunohistochemistry protocol. Images (2–3 per animal) were quantified using ImageJ.

### RNA-scope protocol

We utilized RNA-scope Multiplex Fluorescent v2 Assays (ACD biosciences, USA), following the manufacturer’s protocol. Brain sections were post-fixed with 4% PFA and washed in PBS, followed by a series of ethanol dehydrations and subsequent rehydrations prior to treatment with hydrogen peroxide, and washed again with water 3 times. Antigen retrieval was done by protease III treatment in PBS for 10 min at RT; slides were then washed twice with PBS. Sections were hybridized with probes (Glutamate, Cat No. 426231; WT1, Cat No. 432711-C2, ACD biosciences) for 2 h at 40°C then washed with wash buffer. Hybridized probes were detected using the RNA-scope Amplification reagents for 30 min at 40°C. Slides were washed and then incubated with Opal dyes (Akoya biosciences, USA) for 15 min at 40°C.

### Electrophysiology

Mice were used for electrophysiology experiments 4–5 weeks after stereotaxic surgery. Four to six mice (3–5 months old) were deeply anesthetized with isoflurane and decapitated. The brain was rapidly removed and chilled in cutting artificial Cerebrospinal Fluid (ACSF) containing (in mM): N-methyl-D-glucamine 93, HCl 93, KCl 2.5, NaH_2_PO_4_ 1.2, NaHCO_3_ 30, HEPES 20, glucose 25, sodium ascorbate 5, thiourea 2, sodium pyruvate 3, MgSO_4_ 10, and CaCl_2_ 0.5, pH 7.4. The brain was embedded in 2% agarose and coronal slices (200 μm thick) were made using a Compresstome (Precisionary Instruments, USA). Brain slices were allowed to recover at 33 ± 1°C in ACSF solution for 30 min and thereafter at room temperature in holding ACFS, containing (in mM): NaCl 92, KCl 2.5, NaH_2_PO_4_ 1.2, NaHCO_3_ 30, HEPES 20, glucose 25, sodium ascorbate 5, thiourea 2, sodium pyruvate 3, MgSO_4_, and CaCl_2_ 2, pH 7.4. After at least 1 h of recovery, the slices were transferred to a submersion recording chamber and continuously perfused (2–4 mL/min) with ACSF containing (in mM): NaCl 124, KCl 2.5, NaH_2_PO_4_ 1.2, NaHCO_3_ 24, HEPES 5, glucose 12.5, MgSO_4_ 2, and CaCl_2_ 2, pH 7.4. One μM TTX, or 100 μM Gabazine + 1 μM TTX, was added at least 30 min before mIPSC or mEPSC recordings, respectively. All the solutions were continuously bubbled with 95% O_2_/5% CO_2_. CA3 pyramidal cells and interneurons were visually identified with infrared differential contrast optics (BX51; Olympus, Japan). Whole-cell patch-clamp recordings were performed at room temperature using a Multiclamp 700 A amplifier (Molecular Devices, USA). Recording electrodes (3–5 MΩ) pulled from borosilicate glass were filled with solution containing (in mM): Cs-gluconate 122, HEPES 10, KCl 5, MgATP 5, Na_2_GTP 0.5, QX314 1, and EGTA 1, pH 7.25. Lucifer Yellow (25 μM) was added to the pipette solution for post-recording immunohistochemical confirmation of the recorded cells. Data acquisition (filtered at 10 kHz and digitized at 10 kHz) and analysis was performed with pClamp 11 software (Molecular Devices, USA). Mini excitatory postsynaptic currents (mEPSCs) and mini inhibitory postsynaptic currents (mIPSCs) were recorded in voltage-clamp mode at a holding potential of −90 mV and + 10 mV, respectively, for 5–10 min, beginning 3 min following breakthrough. Only cells with stable input resistances were included in the analysis. For neuronal cell excitability measurements, brain slices were perfused with normal ACSF, neurons were current clamped, and 10–15 500-ms current steps in intervals of 10 pA were applied to induce firing. Recording and analysis were conducted in a blinded manner.

### Replication, blinding, and statistical analysis

For behavior experiments, the results were obtained from pulling together animals from two to three different cohorts. The number of replicates is provided in the figure legends. No statistical method was used to predetermine sample sizes, but our sample sizes are in line with those reported in previous publications for similar behavior tests. For all the electrophysiology and behavior experiments, the experimenter was blind to the mice’s genotype. The coding for the different groups was revealed only after the data were pulled together and analyzed. Data are represented as mean ± s.e.m. Data were analyzed using either a two-tailed independent-samples *t*-test or as a one- or two-way ANOVA using GraphPad Prism Version 7.02 (La Jolla, CA). Tukey’s or Bonferroni’s *post-hoc* tests were employed to examine biologically relevant interactions. Data distributions passed the normality test (alpha = 0.05). Symbols denote significant differences between groups: *p < 0.05, **p < 0.01, and ***p < 0.001.

## Results

### Wt1Δ*^DGC^* mice show impaired memory discrimination when the level of interference is high

**FIGURE 1:**
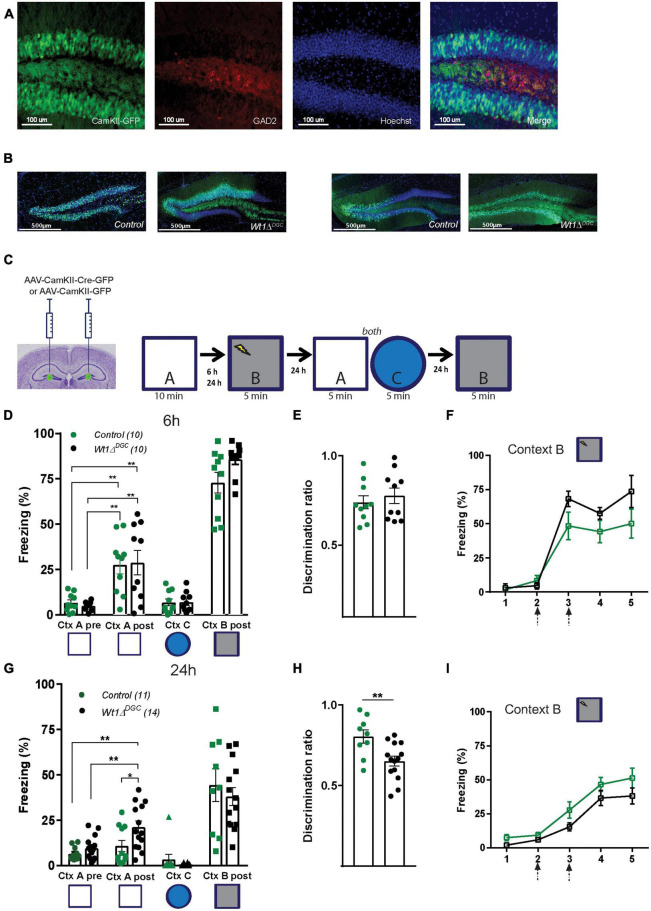
Wt1Δ^DGC^ mice show impaired memory discrimination. **(A)** Representative images showing that the expression of GFP driven by AAV-CamKII promoter does not colocalize with GABAergic neurons stained for GAD2. Scale bar = 100 μm. **(B)** Representative images showing the expression of GFP in the DG in Control and Wt1Δ^DGC^ groups. Scale bar = 500 μm. **(C)** Wt1^fl/fl^ Cre negative mice were injected bilaterally with either AAV8-CamKII-GFP or AAV8-CamKII-Cre-GFP virus (0.5 μL/side) into the DG. Experimental scheme: Post-surgery mice were allowed to explore a neutral Context A, and 6 or 24 h later they were fear conditioned in Context B. Freezing levels were measured in both Context A and the novel Context C and back in the Context B. **(D)** Control and Wt1Δ^DGC^ groups showed increased freezing in the neutral Context A after being shocked in Context B when the exploration time was 6 h (Two-way repeated measures ANOVA, context, *F*(1,9) = 49.95, *p* < 0.001; genotype, *F*(1,9) = 0.002192, *p* < 0.9637; context x genotype, *F*(1,9) = 0.09596, *p* = 0.7638, Bonferroni’s *post-hoc* test: Ctx A, Control vs. Wt1Δ^DGC^ = ns; Ctx A’, Control vs. Wt1Δ^DGC^ = ns). Freezing for Context C (unpaired *t*-test, *p* = 0.9503) and Context B post-shock (unpaired *t*-test, *p* = 0.5186) was similar between groups. **(E)** Both groups displayed a low discrimination ratio (unpaired *t*-test, *p* = 0.5186). **(F)** Learning curve in shock Context B was similar for both groups. **(G)** At 24 h protocol, only Wt1Δ^DGC^ mice showed impaired memory discrimination. Wt1Δ^DGC^ group froze more than the control group in Context A after being fear conditioned in Context B (Two-way repeated measures ANOVA followed by Bonferroni’s *post-hoc* test. Context, *F*(1,13) = 12.01, *p* = 0.0042; genotype, *F*(1,13) = 5.881, *p* = 0.0306; context x genotype, *F*(1,7) = 2.448, *p* = 0.1617, Bonferroni’s *post-hoc* test: Ctx A, Control vs. Wt1Δ^DGC^ = ns; Ctx A’, Control vs. Wt1Δ^DGC^ *p* = 0.019. Freezing for Context C (unpaired *t*-test, *p* = 0.3298) and Context B post-shock (unpaired *t*-test, *p* = 0.5047) were similar. **(H)** The discrimination ratio was statistically significant (unpaired *t*-test, *p* = 0.0072). **(I)** Both groups showed similar learning curves in shock Context B. Arrows in panels **(F,I)** indicate shock delivery. Values are shown as mean ± s.e.m. Results are from two independent experiments. Statistical significance is denoted by *p < 0.05, **p < 0.01.

To study the precise contribution of WT1 in memory interference, we selectively ablated WT1 in DGCs (Wt1Δ*^DGC^*). To do so, we bilaterally injected AAV-CamKII-Cre in the DG of Wt1*^fl/fl^* animals, allowing for the ablation of WT1 under the Ca^2+^/calmodulin-dependent protein kinase II (CamKII) promoter which is abundantly expressed in DG excitatory glutamatergic cells (Wang et al., 2013), and avoiding deletion in the local GABAergic neurons (Dimidschstein et al., 2016; Figures 1A, B). Wt1Δ*^DGC^* mice were then trained in a context discrimination test which included a within-test comparison of lower and higher interference memories. Because any behavior presumably is subject to some level of interference, what represents a low versus high interference task can be viewed only in relative terms. Animals were first exposed to non-shock neutral Context A and then fear conditioned in the shock Context B (in WT mice, learning increased the expression of WT1 RNA in the DG, data not quantified, Supplementary Figure 1). We measured freezing behavior in the non-shock neutral Context A before and after mice were exposed to the shock Context B, and calculated the discrimination ratio [(Freezing Context A)/(Freezing Context A + Freezing Context B)] as an index of memory discrimination over memory generalization. Contexts A and B were similar to create a high level of interference. Animals were then placed back in Context A and into the highly dissimilar Context C. Then 24 h later, mice were placed back in the shock Context B (Figure 1C). We found that with 6 h separating the two similar contexts A and B, both Control and Wt1Δ*^DGC^* mice displayed high levels of freezing in the neutral context (“A”) after being shocked (“B”), suggesting incomplete memory discrimination. The training-induced memory did not generalize to the dissimilar Context C, as freezing levels for both groups were in the baseline range (Figure 1D). Both groups showed similar values for discrimination ratio (Figure 1E) and a comparable percentage of freezing during training in shock Context B (Figure 1F). In a separate cohort of mice, exposure to Context A and B was performed 24 h apart. While the control group was capable of accurate discrimination, freezing levels for the Wt1Δ*^DGC^* group in Context A after fear conditioning were higher than baseline levels for the same context before fear conditioning (Figure 1G). Importantly, both groups recognized Context C (Figure 1G). Impaired memory discrimination was further evidenced by a lower discrimination ratio for the Wt1Δ*^DGC^* group compared to controls (Figure 1H). Both groups showed comparable learning curves during training in shock Context B (Figure 1I).

**FIGURE 2:**
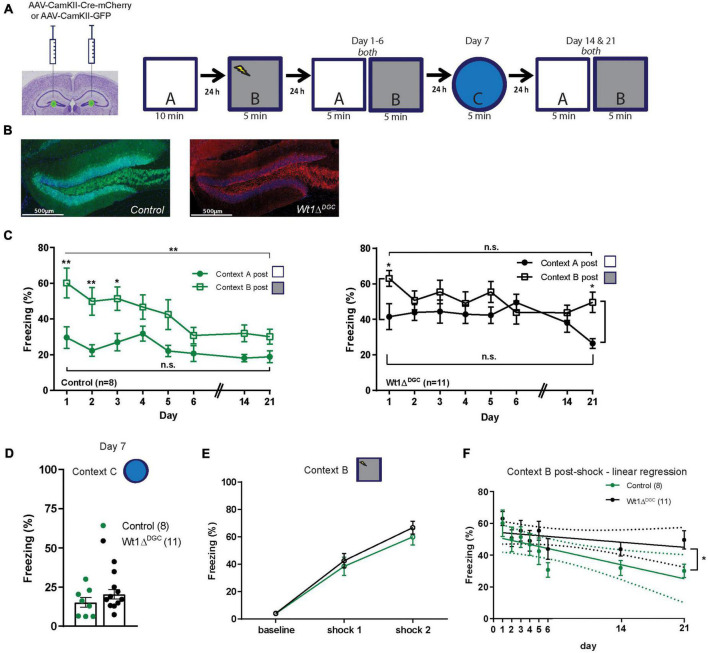
Long-term effect on memory discrimination in Wt1Δ^DGC^ mice. **(A)** Wt1^fl/fl^ Cre negative mice were injected bilaterally with either AAV8-CamKII-GFP or AAV8-mCherry-Cre-GFP virus (0.5 μL/side) into the DG. Experimental scheme: Post-surgery mice were allowed to explore the neutral Context A, and 24 h later they were fear conditioned in Context B. Freezing levels were then measured for both Contexts A and B for 6 consecutive days, in a new Context C on day 7, and then back to Contexts A and B on days 14 and 21. **(B)** Representative images showing GFP or mCherry expression in DG. Scale bar = 500 μm. **(C)** Comparison of the percentage of freezing between the two highly similar Contexts A and B. Left: Control group showed well-separated curves for Contexts A and B indicating memory discrimination (two-way ANOVA RM, main effect of day, *F*_(7_,_98)_ = 7.665, *p* < 0.0001; main effect of context, *F*_(1_,_14)_ = 11.08, *p* = 0.0050; day x context interaction, *F*_(7_,_98)_ = 1.941, *p* = 0.0711; Bonferroni’s *post-hoc* test: day 1 *p* = 0.0012, day 2 *p* = 0.0044, day 3 *p* = 0.0167; unpaired *t*-test comparing day 1 vs. day 21: Context B, *p* = 0.0061, Context A *p* = 0.1383). Right: In contrast, freezing curves for the Wt1Δ^DGC^ group for shock and neutral contexts were similar indicating poor memory discrimination (two-way ANOVA RM, main effect of day, *F*_(7_,_160)_ = 1.449, *p* = 0.1894; main effect of context, *F*_(1_,_160)_ = 13.86, *p* = 0.003; day x context interaction, *F*_(7_,_160)_ = 1.427, *p* = 0.1979, Bonferroni’s *post-hoc* test: day 1 *p* = 0.0492, day 21 *p* = 0.0269; unpaired *t*-test comparing day 1 vs. day 21: Context B, *p* = 0.0802, Context A *p* = 0.0706). **(D)** Although not significant, both groups showed an increase in freezing for the novel dissimilar context C (unpaired *t*-test *p* = 0.2531) and **(E)** similar learning curves in shock context B. **(F)** Linear Regression modeling freezing behavior for Context B post-shock. Differences between the elevations were significant, *p* = 0.0336. Values are shown as mean ± s.e.m. Results are from two independent experiments. N.s. not significant. Statistical significance is denoted by *p < 0.05, **p < 0.01.

We then tested the persistence of impaired discrimination in Wt1-deficient mice. Wt1*^fl/fl^* mice were injected with either AAV-CamKII-Cre-mCherry or AAV-CamKII-GFP virus into the DG (Figure 2A) and were trained in an extended version of the context discrimination test protocol where they were first exposed to Context A, and fear-conditioned in Context B 24 h after. Two days later they were randomly placed back into the overlapping Contexts A and B for 6 consecutive days, in the novel Context C on day 7, and then back to Contexts A and B on days 14 and 21 (Figure 2B). Confirming what we observed in the previous experiment, Wt1Δ*^DGC^* mice were deficient in discriminating between the two highly similar contexts (Figure 2C). Both groups showed freezing in the novel Context C, suggesting memory generalization 7 days after being shocked in Context B (Figure 2D). This result is consistent with evidence that contextual memories become less precise and tend to generalize over time (Guo et al., 2018). The learning curve was comparable between groups (Figure 2E). Further analyses of freezing behavior in shock Context B demonstrated that the Wt1Δ*^DGC^* mice had an impairment in fear memory extinction (Figure 2F). Lastly, we tested the effect of WT1 ablation in CA1 pyramidal neurons (Wt1Δ^*CA*1^, Supplementary Figure 2A) in the contextual discrimination test (Supplementary Figure 2B). Both groups behaved similarly and freezing time in Context A was higher than baseline freezing in the same context before animals were fear-conditioned in Context B (Supplementary Figure 2C). Similar discrimination ratio values were found for both groups (Supplementary Figure 2D). It is known that the CA1 region is involved in the recall of overlapping memories (Cai et al., 2016) and WT1 ablation in CA1 alters synaptic efficiency in this region (Mariottini et al., 2019), and additional studies need to be performed to better understand the role of WT1 in memory interference in CA1. Groups were able to recognize a non-overlapping Context C (Supplementary Figure 2E), had comparable freezing levels in the shock Context B (Supplementary Figure 2F), and showed similar learning curves (Supplementary Figure 2G). Overall, our behavioral data suggest that impaired memory discrimination in Wt1Δ*^DGC^* mice can be attributed to two factors: increased freezing in the neutral Context A and impaired memory extinction for shock Context B. These results demonstrate that WT1 expression is involved in the precise formation of similar overlapping contextual memories in the hippocampus dentate gyrus.

### WT1 ablation increases excitability in the DGCs and alters the synaptic properties of the DG → CA3 feedforward input

**FIGURE 3:**
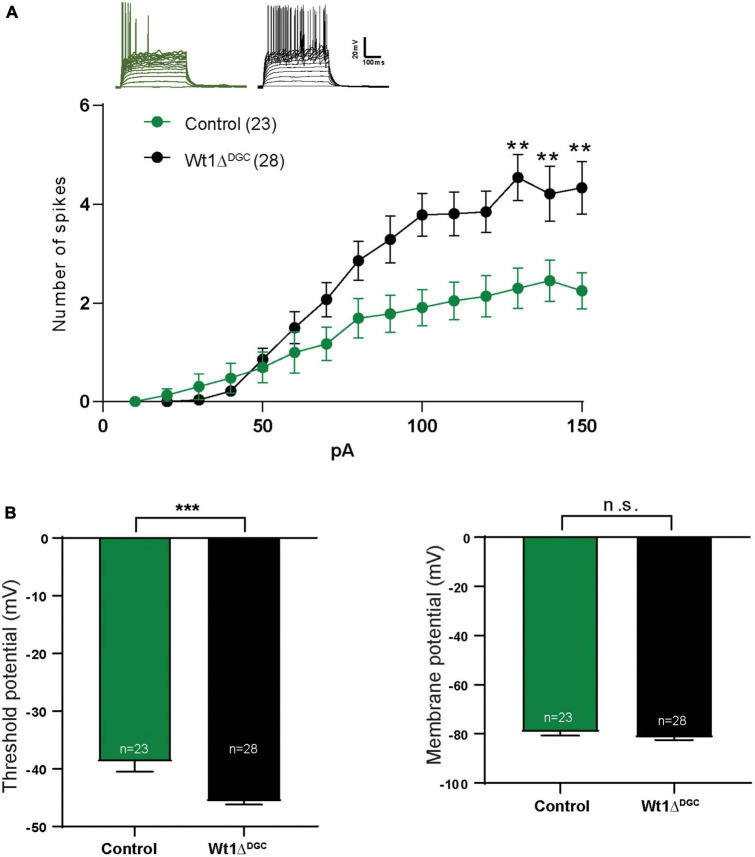
WT1 ablation in the DG granule cells increases excitability. Whole-cell patch recordings in mice injected either with AAV8-CamKII-GFP (Control) or AAV8-CamKII-Cre-GFP virus (Wt1Δ^DGC^) into the DG and tested 4 weeks later. **(A)** Representative traces from both groups. With the increasing of injected current, the number of spikes also increased in the Wt1Δ^DGC^ group compared to controls (*p* < 0.01). **(B)** Left: Granule cell threshold potential decreased (*p* = 0.0001) in the Wt1Δ^DGC^ mice compared to the control group without changing the membrane potential (*p* = 0.1803, right panel). Values are shown as mean ± s.e.m. n.s.: not significant. Two-way repeated measures ANOVA; Control group, *n* = 5, 23 cells patched; Wt1Δ^DGC^ group; *n* = 5, 28 cells patched. Statistical significance is denoted by **p < 0.01, ***p < 0.001.

**FIGURE 4:**
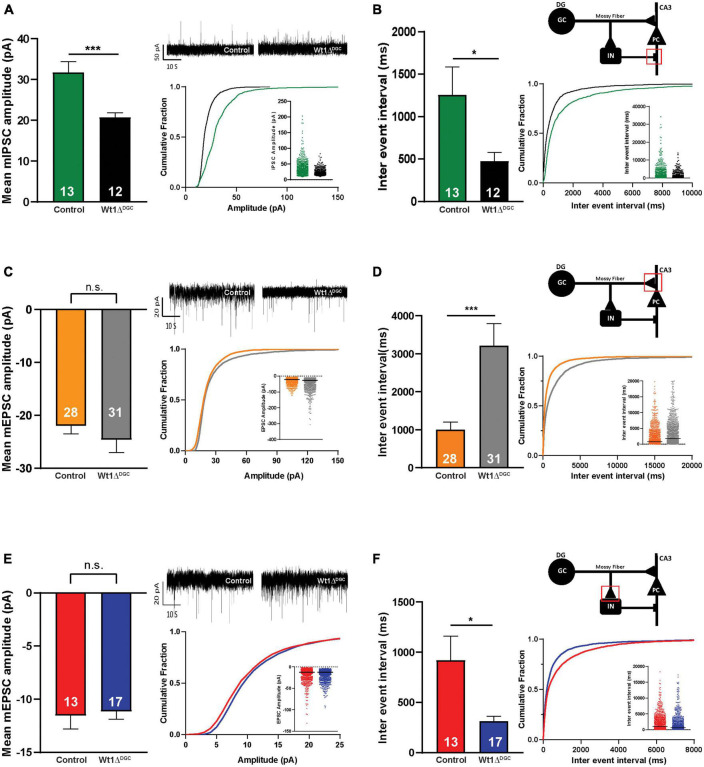
Effects of WT1 ablation in granule cells on synaptic properties of the DG-CA3 circuit. **(A)** Left: Amplitude of the inhibitory synapses (mIPSCs) recorded in CA3 pyramidal cells was decreased (*p* = 0.0009) in Wt1Δ^DGC^ mice compared to controls. Right: Representative traces, cumulative curves, and scatter plots for mIPSCs amplitude of both groups. **(B)** Left: Inter-event interval was decreased (*p* = 0.0381) in Wt1Δ^DGC^ mice. Right: Cartoon indicating recorded synapse. Cumulative curves and scatter plots for mIPSCs inter-event interval of both groups. 13 cells patched for the control group and 12 cells patched for Wt1Δ^DGC^ group. **(C)** Left: Amplitude of excitatory synapses (mEPSCs) in CA3 pyramidal cells were not changed (*p* = 0.3697). Right: Representative traces, cumulative curves, and scatter plots for mEPSCs amplitude of both groups. **(D)** Left: Inter-event interval was increased (*p* = 0.001) in Wt1Δ^DGC^ mice. Right: Cartoon indicating recorded synapse. Cumulative curves and scatter plots for mEPSCs inter-event interval of both groups. A total of 28 cells patched for the control group and 31 cells patched for Wt1Δ^DGC^ group **(E)** Left: The amplitude of the excitatory synapses (mEPSCs) recorded in interneurons was unchanged (*p* = 0.7953) in Wt1Δ^DGC^ mice compared to controls. Right: Representative traces, cumulative curves, and scatter plots for mEPSCs amplitude of both groups. **(F)** Left: Inter-event interval was decreased (*p* = 0.0165) in Wt1Δ^DGC^ group. Right: Cartoon indicating recorded synapse. Cumulative curves and scatter plots for mEPSCs inter-event interval of both groups. A total of 13 cells patched for the control group and 17 cells patched for Wt1Δ^DGC^ group. Bar graphs show summary data, with numbers of cells patched indicated. Values are shown as mean ± s.e.m. n.s.: not significant. Unpaired *t*-test; statistical significance is denoted by *p < 0.05, ***p < 0.001.

We investigated the impact of WT1 ablation on DGC excitability with whole-cell patch-clamp electrophysiological recordings from granule cells in control and Wt1Δ*^DGC^* mice. DGCs of the Wt1Δ*^DGC^* group were more excitable than controls, as shown by an increase in the maximum firing rate in response to current injection (Figure 3A) and a decrease in the spike threshold potential (Figure 3B, left), with no effect on the membrane resting potential (Figure 3B, right). These results confirm the previous report showing that ablation of WT1 levels in CA1 results in increased excitability (Mariottini et al., 2019). Within the DG → CA3 circuit, activity in DGCs is conveyed to CA3 pyramidal cells (PC) through (i) direct excitation by the mossy fibers, and (ii) feedforward inhibition via interneurons. A balance between these excitatory and inhibitory inputs (E/I) to the PC is critical for precision in memory formation and retrieval (Ruediger et al., 2011; Guo et al., 2018). Our findings showing increased DGC excitability prompted us to investigate whether this change would disrupt the normal E/I balance in the DG → CA3 circuit and facilitate excitatory transmission. We recorded miniature (mini) excitatory and inhibitory postsynaptic currents (mEPSCs and mIPSCs, respectively) in CA3 pyramidal cells, and mEPSCs in interneurons (Supplementary Figure 3B), giving us information regarding the intrinsic activity of all three of the major synapses of the DG → CA3 circuit. We found that mIPSC amplitudes recorded in CA3 PCs were significantly depressed (Figure 4A) in Wt1Δ*^DGC^* mice compared to the control group. However, mEPSC amplitude was not affected by WT1 ablation, either in CA3 PCs (Figure 4C) or interneurons (Figure 4E). WT1 ablation in DGCs also had effects on mini frequencies at synapses on CA3 pyramidal cells (Figures 4B, D) and in interneurons (Figure 4F), suggesting that there may be effects of prolonged WT1 ablation on intrinsic, non-evoked transmitter release within the circuit (see Section “Discussion”). Together, our results suggest that WT1 ablation in DGCs reduces feedforward inhibition mediated by the interneurons in the DG → CA3 circuit, evidenced by reduced inhibitory input to CA3, consistent with a pro-excitatory shift in E/I balance.

### Silencing DGCs via inhibitory DREADDs rescues memory discrimination impairment

**FIGURE 5:**
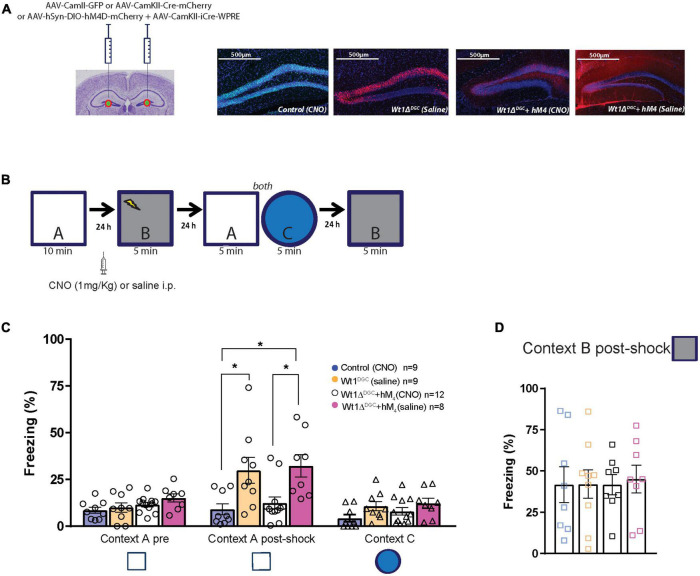
Silencing granule cells by activation of M_4_ DREADD receptors rescues memory discrimination impairment. **(A)** Mice were injected with a mix of AAV8-hSyn-DIO-hM4D(Gi)-mCherry + AAV8-CamKII(0.4)-iCRE-WPRE or AAV8-CamKII-Cre-mCherry or AAV8-CamKII-GFP into the DG. Representative examples of GFP and mCherry virus expression in the DG. Scale bar = 500 μm. **(B)** Behavior scheme: Mice were allowed to explore Context A and in the next day, CNO or Saline was injected 45 min before animals were fear-conditioned in Context B (syringe). Freezing behavior was then assessed the next day in Context A and Context C, and 24 h later they were placed back in Context B. **(C)** All groups showed comparable freezing time in neutral Context A. However, after had been shocked in Context B, Wt1Δ^DGC^ mice treated with saline showed impaired memory discrimination which was rescued in the group treated with CNO [one-way ANOVA multiple comparisons, *F* = 5.819, *p* = 0.0025, Turkey’s *post-hoc*: Control vs. Wt1Δ^DGC^ (Sal-treated) *p* = 0.0265; Control vs. Wt1Δ^DGC^ + hM4D (CNO-treated) *p* = 0.0143; Wt1Δ^DGC^ + hM4D (CNO-treated) vs. Wt1Δ^DGC^ + hM4D (Sal-treated) *p* = 0.02920]. The effect did not generalize to Context C (one-way ANOVA multiple comparisons, *F* = 2.197, *p* = 0.1063). **(D)** All groups showed comparable freezing behavior in the shock Context B (one-way ANOVA, *F* = 0.0365, *p* = 0.9910). Values are shown as mean ± s.e.m. Results are from three independent experiments. Statistical significance is denoted by *p < 0.05.

The experiments thus far indicate that decreased levels of WT1 in DGCs lead to their activation and a loss of pattern separation. Hence, reduction of DGC activity should rescue the memory discrimination deficit caused by WT1 ablation. To test this hypothesis, Wt1*^fl/fl^* mice were injected with a mix of AAV8-hSyn-DIO-hM4D(Gi)-mCherry + AAV8-CamKII(0.4)-iCRE-WPRE viruses or control virus. This strategy allowed us to express the hM4D DREADD inhibitory receptor in DGCs where WT1 was knocked down (mCherry Cre + cells, Figure 5A). We used this DREADD approach to decrease the excitability caused by WT1 ablation. Clozapine-N-oxide (CNO) activates hM4D DREADD receptors, and in this case, inhibits neurons that express them (Supplementary Figure 4; López et al., 2016). Mice were trained in the context discrimination test protocol, but this time they received an i.p. injection of CNO (1 mg/kg) or saline 45 min before being placed in the shock Context B (Figure 5B). Silencing the DGCs rescued the behavior phenotype in Wt1Δ*^DGC^* mice, with freezing levels for Context A post-shock comparable to the control group treated with saline (Figure 5C). This approach did not affect the ability to discriminate a different Context C (Figure 5C), nor freezing behavior in the shock Context B (Figure 5D). Taken together, these findings, combined with our electrophysiology experiments, indicate that the increased excitability of DGCs and reduced feedforward inhibition to CA3 leads to excessive stimulation of CA3 during memory formation, reducing the capability of the hippocampus to separate two overlapping sets of inputs.

## Discussion

Mechanisms that link or separate memories are critically important for organizing related memories stored throughout our lifetime. While the functional and physiological mechanisms underlying the processing, integration, and recall of sensory information have been extensively investigated, the precise and selective molecular mechanisms driving memory discrimination remain elusive. In our study, using viral and transgenic approaches, we first identify that selective WT1 deletion in DGCs disrupts memory discrimination. We then show that WT1 deletion is associated with physiological effects on DGC excitability and on synaptic transmission in the DG circuit. Finally, using a chemogenetic approach, we establish a causal link between DGC hyperexcitability and memory discrimination impairments. Together this study provides new information on the role of the transcriptional repressor WT1 in modulating DG feedforward inhibition onto CA3 pyramidal neurons to improve memory precision, therefore reducing interference and facilitating accurate storage and retrieval of daily similar episodes (Rolls, 2013; Nakazawa, 2017). It is well accepted that DG-mediated pattern separation supports differential memory encoding for similar memories (Treves et al., 2008; Baker et al., 2016). Several behavioral studies (McHugh et al., 2007; Kheirbek et al., 2012; Rangel et al., 2014; Yokoyama and Matsuo, 2016; Morales et al., 2021) have demonstrated that when DG function is impaired, animals generalize fear toward a neutral non-shock context, suggesting that DG mossy fiber output is necessary for context discrimination and promoting the separation of context-encoding CA3 ensembles, rather than simply establishing the context-fear association memory. In agreement with these studies, our behavioral data showed that mice in which WT1 was ablated in DG granule cells showed impaired context discrimination when the level of interference was high but were able to recognize a low interference context without compromising fear-conditioned learning. More recent studies analyzing memory linking and engram formation in the amygdala (Rashid et al., 2016) and the CA1 region (Cai et al., 2016) demonstrated that two similar events that occur in close temporal proximity can be linked by co-allocation of neurons to overlapping engrams, a phenomenon that is presumably enhanced by high excitability in the circuit, in agreement with accumulating evidence that excitability and functional connectivity contribute to memory interference (Silva et al., 2009; Rogerson et al., 2014). We found that DGCs in the Wt1Δ^DGC^ mice were more excitable than in the control group and that selectively inhibiting those cells where WT1 was ablated was sufficient to rescue the impaired memory discrimination observed in the Wt1Δ^DGC^ group. Taken together the data suggest that excitability in DGCs modifies the circuit level computation by altering the ability of the inhibitory synapses between interneurons and CA3 pyramidal cells that drives pattern separation and memory discrimination to establish a precise memory in the CA3 region. Future experiments will be required to elucidate the molecular mechanism by which changes in WT1 levels in the granule cells control the output of the interneurons between mossy fibers and CA3 pyramidal cells and to confirm whether engrams in the dentate gyrus co-allocate and overlap.

As information flows within the hippocampal formation, inhibition refines the excitatory interactions between subfields. In the DG → CA3 circuit, GABAergic feedforward inhibition is critical for shaping CA3 activation patterns by determining the temporal window for pyramidal cell firing (Mori et al., 2004; Guo et al., 2018). It is estimated that the mossy fibers make 50× more synaptic contacts onto these GABAergic interneurons than onto pyramidal cells (Acsády et al., 1998), and the strength of feedforward inhibition connectivity by mossy fiber filopodial growth in CA3 correlates with the establishment of precise memory for hippocampal spatial tasks (Ruediger et al., 2011). In our study, WT1 ablation in DGC had complex effects on synaptic transmission in this circuit. Particularly striking was the differential effect on responsivity to excitatory and inhibitory events. The amplitudes of mEPSCs at both interneurons and CA3 neurons were unaffected by WT1 ablation, suggesting that expression of postsynaptic AMPARs at both sets of synapses does not depend on WT1 in DGCs. However, in CA3 neurons the loss of WT1 apparently reduced the expression or function of GABARs, since mIPSC amplitude in these neurons was depressed by 35%. Together, these effects of WT1 ablation are expected to render the circuit more excitable, and the observed increase in the intrinsic excitability of DGCs would amplify this bias toward excitation in area CA3 (McBain, 2008). Such a shift in favor of excitation of CA3 pyramidal cells is predicted to decrease memory precision (Mori et al., 2004; Park et al., 2016). Of note, we also found changes in mEPSC and mIPSC frequencies, with increased frequency at both sets of synapses in the feedforward inhibitory branch (DGC → interneuron and interneuron → CA3) and decreased frequency in the excitatory branch (DGC→CA3). If these changes, which were detected in the presence of tetrodotoxin, were entirely due to altered basal (non-evoked) release probabilities at pre-existing synapses [for example, due to effects on presynaptic calcium handling (Sharma and Vijayaraghavan, 2003; Kwon and Castillo, 2008)], they might have limited relevance in an active circuit where most transmitter release is in response to action potentials. Alternatively, if increases or decreases in synapse number contribute to the observed effect of WT1 ablation on mini frequencies, then the postsynaptic bias toward excitation might be offset to some degree. While our analysis focused on several key synapses in the DG → CA3 circuit, it is important to note that other synapses within this circuit are subject to forms of plasticity, including synapses from entorhinal cortex → CA3, CA3 → interneuron, and interneuron → interneuron. The sustained depletion of WT1 in the DGCs (we recorded 4–5 weeks after surgery) and the corresponding long-term increase in DGC excitability would be expected to induce adaptive changes in the downstream circuitry of the dentate gyrus. Thus, it is likely that reduced memory precision following WT1 ablation in DGCs reflects circuit-level changes in synaptic weight and neuronal excitability, including but not limited to the specific synapses and cells that we studied.

Overall, our data indicate that WT1 ablation in the DG granule cells causes an increase in intrinsic excitability which, in turn, affects neuronal computation in the DG → CA3 circuit and disrupts pattern separation, causing memory interference. The reduction in the inhibitory input to CA3 and consequent E/I imbalance would exacerbate the recruitment of CA3 by the hyperexcitable DGCs, potentially degrading the ability of the circuit to properly separate memories. We suggest that WT1 maintains the E/I balance of synaptic transmission by strengthening the mossy fiber filopodia synapse onto CA3 interneurons, thus avoiding over-activation of CA3 pyramidal neurons. Maintaining DGC → interneuron connectivity might, in turn, maintain the engram in the DG, thereby promoting stabilization of memory traces and reducing interference between similar engrams (Guo et al., 2018). Factors that regulate destabilization of branched F-actin networks in the mossy fiber pathway may dictate the number of mossy fibers filopodial contacts with interneurons to promote inhibition onto CA3 in response to learning. Further studies are needed to clarify the molecular pathways underlying the mechanisms through which WT1 controls the mossy fiber filopodia synapse onto interneurons and its contribution to network dynamics during and after behavioral learning.

## Significant statement

To prevent interference between memories that share similar features, the brain needs to store distinct electrical activity patterns. This ability critically depends on the hippocampus, and it is mediated by a process called pattern separation which is still poorly understood. WT1’s function in normal brain physiology and memory is still unknown. Our new findings support a critical role for WT1 in normal brain physiology and cognition and provide new information on WT1 effects in the dentate gyrus, and its role in modulating discrimination between similar events that occur close in time. Our results demonstrate the importance of cell-level changes in modulating intercellular functions to control organismal behavior–in this case, memory precision.

## Data availability statement

The original contributions presented in this study are included in the article/Supplementary material, further inquiries can be directed to the corresponding author.

## Ethics statement

The animal study was approved by the Institutional Animal Care and Use Committee (IACUC) at the Icahn School of Medicine at Mount Sinai. The study was conducted in accordance with the local legislation and institutional requirements.

## Author contributions

LM, VP, CM, RB, and RI conceived and designed the experiments. LM, VP, and CM performed the experiments. LM, VP, and NJ analyzed the data. SP provided the technical support. LM, VP, RB, and RI contributed to the writing and editing of the manuscript. All authors read and approved the final version of the manuscript.

## Supplementary material

Supplementary Figure 1 WT1 RNA expression increases with learning. Representative images showing WT1 RNA expression in the DG. Scale bar = 40 μm. Wildtype mice were divided in two groups: one group was exposed to context-only **(upper panel)** and the second group was exposed to context-paired to footshock (**bottom panel**, 2 shocks, 0.65 mA, 2 s, 1 min apart). Animals were sacrificed 2 h later and stained for WT1 (red), glutamate (green) and DAPI (blue) following the manufacturer protocol (Advanced Cell Diagnostics).

Supplementary Figure 2 WT1 ablation in the CA1 region does not impair memory discrimination. **(A)** Wt1^fl/fl^ Cre negative mice were injected bilaterally with either AAV8-CamKII-GFP or AAV8-CamKII-Cre-GFP virus (0.5 μL/side) into the CA1 and tested 4 weeks later. Representative images showing the expression of GFP in the CA1 in both groups. Scale bar = 40 μm. **(B)** Experimental scheme: Post-surgery mice were allowed to explore a neutral Context A, and 24 h later they were fear conditioned in Context B. Freezing levels were then measured in both the Context A (high interference context) and the novel Context C (low interference context) and back again in the shock Context B. **(C)** A comparison of freezing behavior for Context A before and after fear conditioning showed no difference between groups suggesting that mice were capable to discriminate between the two similar contexts (One-way ANOVA, *F* = 2.682, *p* = 0.0695). **(D)** Both groups showed a similar discrimination ratio (unpaired t-test, *p* = 0.8661). **(E)** Mice from both groups were capable to recognize a new Context C (unpaired t-test, *p* = 0.4854) and **(F)** showed high levels of freezing for shock Context B, as expected. **(G)** Learning curves for both groups was similar. Values are shown as mean ± s.e.m. n.s.: not significant.

Supplementary Figure 3 **(A)** Representative agarose gel showing PCR genotyping of flox/flox (∼230 bp) Cre-negative mice. **(B)** Patched CA3 Interneuron. Representative image showing patched interneuron stained with Lucifer Yellow. Arrows indicate GAD2 staining. Scale bar = 40 μm.

Supplementary Figure 4 Activation of M_4_ DREADD receptors by CNO decreases the expression of c-Fos+ cells in DG. **(A)** Representative images showing c-Fos+ cells in the DG. Scale bar = 40 μm. **(B)** Animals treated with CNO showed significantly less c-Fos+ cells compared to the saline-treated group (*t*-test, *p* = 0.0225). Values are shown as mean ± s.e.m. Results are from a pilot experiment. All images were included in the analysis. Statistical significance is denoted by **p* < 0.05.
