# Supplementary figures and images for: Memory discrimination is promoted by the expression of the transcription repressor WT1 in the dentate gyrus
Source: Front Behav Neurosci. 2023 Sep 27;17:1130840. doi: 10.3389/fnbeh.2023.1130840 (PMC10564998; doi:10.3389/fnbeh.2023.1130840)

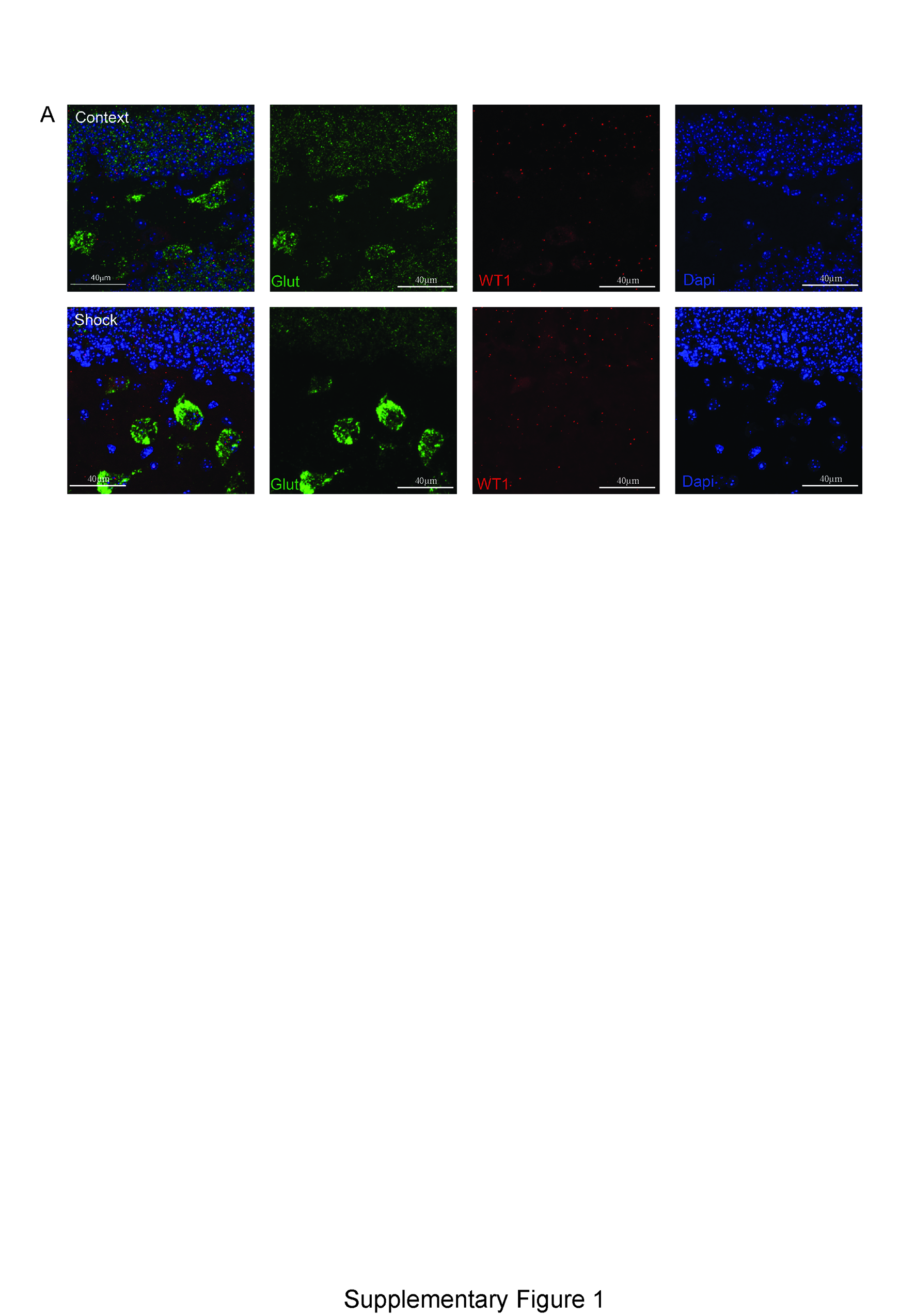

Supplement: Supplementary Figure 1 — WT1 RNA expression increases with learning. Representative images showing WT1 RNA expression in the DG. Scale bar = 40 μm. Wildtype mice were divided in two groups: one group was exposed to context-only (upper panel) and the second group was exposed to context-paired to footshock (bottom panel, 2 shocks, 0.65 mA, 2 s, 1 min apart). Animals were sacrificed 2 h later and stained for WT1 (red), glutamate (green) and DAPI (blue) following the manufacturer protocol (Advanced Cell Diagnostics). [file Image_1.tif]

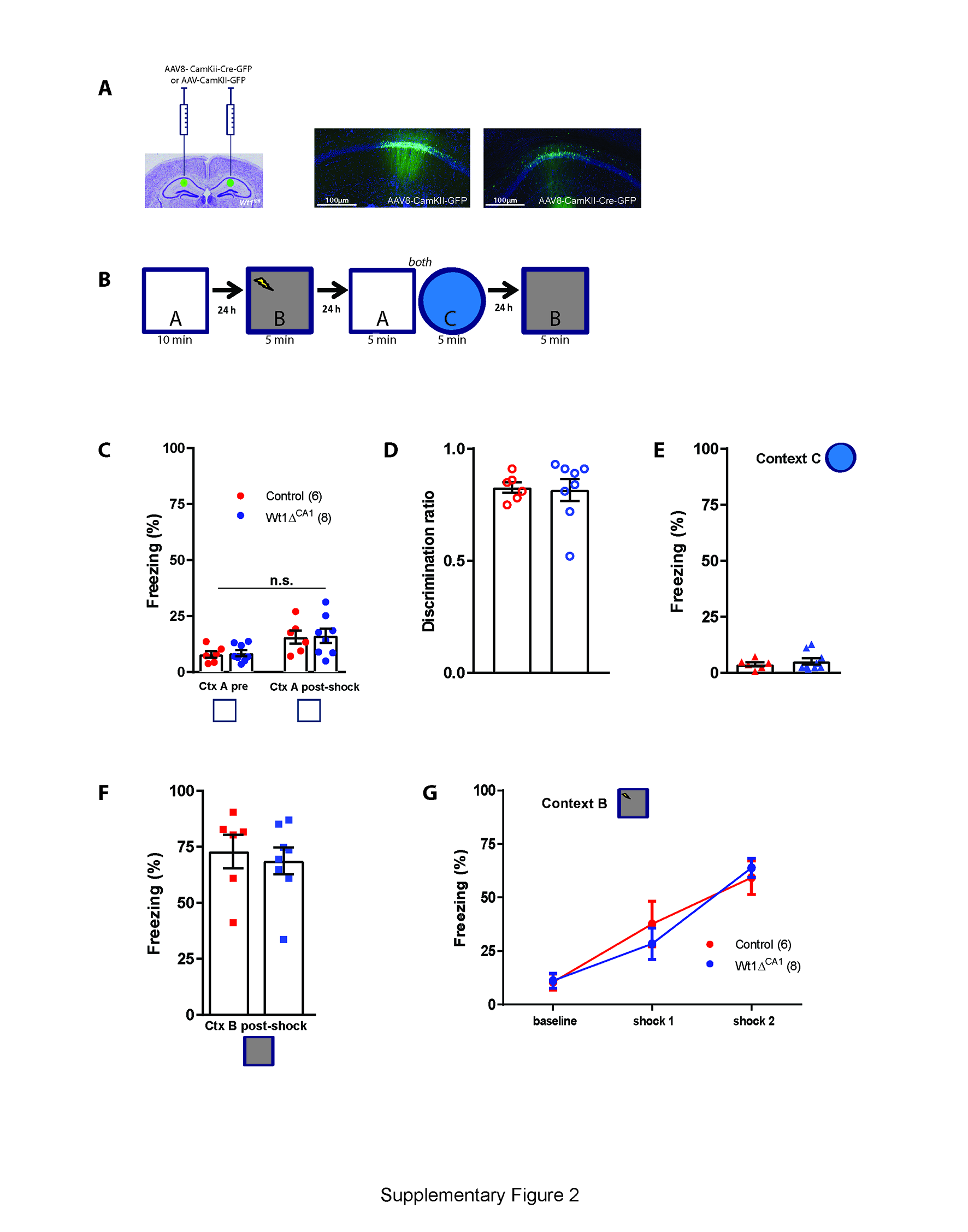

Supplement: Supplementary Figure 2 — WT1 ablation in the CA1 region does not impair memory discrimination. (A) Wt1fl/fl Cre negative mice were injected bilaterally with either AAV8-CamKII-GFP or AAV8-CamKII-Cre-GFP virus (0.5 μL/side) into the CA1 and tested 4 weeks later. Representative images showing the expression of GFP in the CA1 in both groups. Scale bar = 40 μm. (B) Experimental scheme: Post-surgery mice were allowed to explore a neutral Context A, and 24 h later they were fear conditioned in Context B. Freezing levels were then measured in both the Context A (high interference context) and the novel Context C (low interference context) and back again in the shock Context B. (C) A comparison of freezing behavior for Context A before and after fear conditioning showed no difference between groups suggesting that mice were capable to discriminate between the two similar contexts (One-way ANOVA, F = 2.682, p = 0.0695). (D) Both groups showed a similar discrimination ratio (unpaired t-test, p = 0.8661). (E) Mice from both groups were capable to recognize a new Context C (unpaired t-test, p = 0.4854) and (F) showed high levels of freezing for shock Context B, as expected. (G) Learning curves for both groups was similar. Values are shown as mean ± s.e.m. n.s.: not significant. [file Image_2.tif]

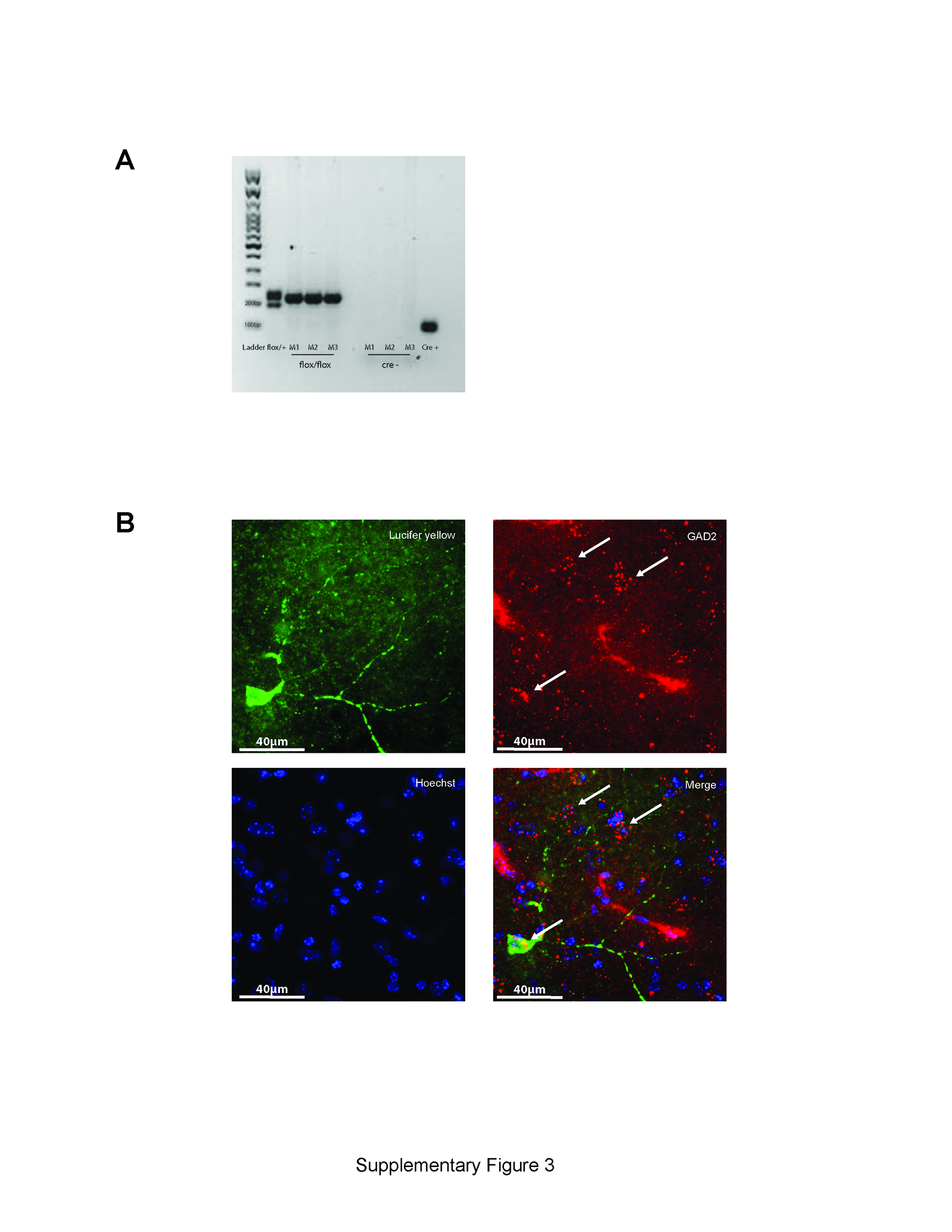

Supplement: Supplementary Figure 3 — (A) Representative agarose gel showing PCR genotyping of flox/flox (∼230 bp) Cre-negative mice. (B) Patched CA3 Interneuron. Representative image showing patched interneuron stained with Lucifer Yellow. Arrows indicate GAD2 staining. Scale bar = 40 μm. [file Image_3.TIF]

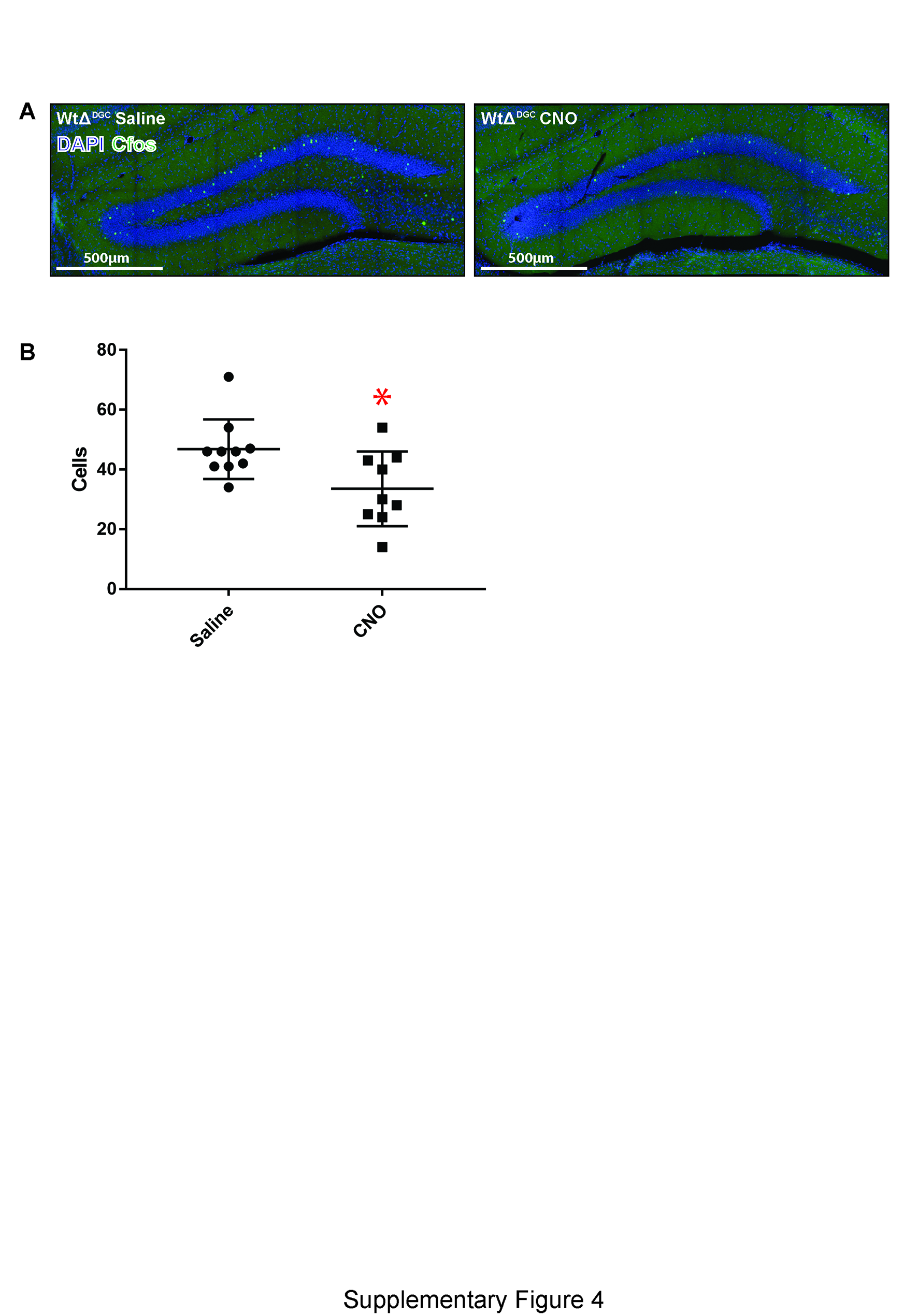

Supplement: Supplementary Figure 4 — Activation of M4 DREADD receptors by CNO decreases the expression of c-Fos+ cells in DG. (A) Representative images showing c-Fos+ cells in the DG. Scale bar = 40 μm. (B) Animals treated with CNO showed significantly less c-Fos+ cells compared to the saline-treated group (t-test, p = 0.0225). Values are shown as mean ± s.e.m. Results are from a pilot experiment. All images were included in the analysis. Statistical significance is denoted by *p < 0.05. [file Image_4.tif]
